# Beyond Efficacy: The Depth and Diversity of Current Internet Interventions

**DOI:** 10.2196/jmir.2206

**Published:** 2012-06-29

**Authors:** Helen Christensen, Alison L Calear, Gerhard Andersson, Frances P Thorndike, Robert J Tait

**Affiliations:** ^1^Black Dog InstituteUniversity of New South WalesSydney, NSWAustralia; ^2^Centre for Mental Health ResearchThe Australian National UniversityCanberraAustralia; ^3^Department of Behavioural Sciences and LearningLinköping UniversityLinköpingSweden; ^4^Department of Psychiatry and Neurobehavioral SciencesUniversity of Virginia Health SystemCharlottesville, VAUnited States

This issue of the *Journal of Medical Internet Research* (JMIR) brings together nine papers selected from presentations at the 5^th^ International Conference of the *International Society for Research on Internet Interventions (ISRII*) held in Sydney, Australia in 2011. The papers highlight the depth and diversity of our field and reflect some of the current major research themes, including issues of stigma, new modes of intervention delivery, tailoring, the role of support, mechanisms of change, target audience considerations, new theoretical approaches, and further grounding in models and frameworks. The field is pushing beyond efficacy trials to better understand when, how, and why Internet interventions work. As was true of many of the presentations at ISRII, this set of papers makes a substantive contribution to our field’s understanding of how to reduce mental and behavioral health problems (for example, depression, anxiety, smoking), improve mental health literacy and knowledge, and encourage help-seeking. In the remaining paragraphs, we summarize several key points on how each paper contributes to the broader research picture in these areas, suggesting future paths of research.

 Stigmatizing attitudes and beliefs towards mental health disorders is a significant problem in the community, with mental health stigma associated with poor help-seeking behavior and treatment adherence. A number of effective programs have been developed to address mental health stigma in the community. These have included programs aimed at challenging stereotypes and increasing knowledge through education and/or personal contact with a person with a mental health problem [1, 2]. The paper by Gulliver et al. [3] presents the efficacy of two brief online interventions to promote help-seeking behavior in elite athletes, with the study suggesting that an online mental health literacy intervention can reduce depression and anxiety stigma. Online interventions such as this one have the potential to reach a broad audience and may be a cost-effective way of reducing stigma in the community. The paper by Farrer and colleagues [4] further supports the effectiveness of online interventions in reducing depression stigma, finding significantly lower levels of stigma amongst participants completing an online cognitive behavior therapy (CBT) program for depression.

 Kauer et al. [5] continued the field’s investigation of using technology to treat depression, but examined the use of mobile phone self-monitoring in a group of adolescents with mild depressive symptoms. The authors found that self-monitoring increased emotional self-awareness which in turn mediated decreases in depressive symptoms. Monitoring symptoms and activities by means of mobile phone technology is likely to become even more common in research given increased use of smart phones in daily life. Smart phones are also extending the reach of these interventions to patient groups with historically limited Internet access.

 Tailored Internet interventions are also being more readily developed and studied. Rather than offering the same intervention to all users, harnessing the full capabilities of the technology allows these interventions to be tailored to specific user characteristics (for example, symptom level, age). Silfvernagel et al. [6] report data from a controlled trial in which individually tailored, guided cognitive-behavior therapy was tested for persons with panic symptoms. Findings suggest that tailoring the Internet intervention to this patient population was feasible and efficacious, adding to the previous promising findings with large effect sizes [7, 8].

 The role of support in Internet interventions has been discussed over the years, with considerable debate about whether support is necessary to achieve optimal outcomes, and, if so, at what cost [7, 9-14]. Support can be provided via multiple formats (for example, personalized emails, texts, phone calls) and can occur at various points in the intervention. Three papers in this special edition address the issue of support by exploring whether clinician delivered, clinician guided, guided or self-guided interventions are equally effective, as well as for whom and when (see [4, 6, 15]). The trial by Farrer et al. was conducted in a national helpline setting and compared outcomes among participants who received: 1) web-based CBT for depression; 2) web-based CBT plus telephone tracking; 3) weekly telephone tracking only; or 3) neither web CBT nor telephone tracking. In this study, telephone support during the actual treatment did not affect outcome, indicating that the temporal aspects of support and contact need to be further investigated [4]. In the Silfvernagel et al. paper [6] mentioned above, the tailored intervention for panic used online therapist guidance to support users. Overall, the role of support and better definitions of precisely what support entails, warrants further evaluation.

 Anderson and colleagues [15] examined a therapist guided Internet intervention in which adolescents and children with anxiety disorders, as well as their parents, were included. Borrowing a concept from traditional face-to-face psychotherapy, the authors investigated the role of working alliance, a term to describe the collaboration between a patient and a therapist. In line with previous studies on adult patients [16, 17], high ratings of working alliance were found, even equivalent to what was observed in clinic-based treatment. Alliance also predicted adherence and outcome for the adolescents but not for the younger children. The findings from this study highlight the importance of investigating differences and similarities between traditional and Internet interventions, as well as the importance of examining mechanisms of change within Internet interventions.

 The use of Internet interventions as a means of addressing problematic behaviors, including substance use, is now well established. A review by Riper and colleagues [18] found that even a single session achieves effects of a similar magnitude to that found for in-person brief alcohol interventions [19] with multi-session interventions producing significantly larger effects. However, much of the research in this field has utilized university samples that can be expected to have higher levels of computer literacy than the general community [20], leaving the effectiveness of Internet interventions in larger populations open to question. The paper by Muñoz et al. addresses this issue, at least in the context of the cessation of smoking [21]. Having demonstrated the efficacy of the combined Spanish / English language San Francisco Stop Smoking Internet site, the resource was opened to the general public (aged 18 or over). With participants from more than 150 counties, the observed quit rate at 12 months (45%) was higher than the baseline trial and incurred minimal costs. High rates of substance use frequently co-occur with other mental health problems [22]. Although, there have been interventions developed specifically addressing those with comorbid alcohol and depression [23], the paper by Farrer et al. [4] shows significant short-term reductions in hazardous alcohol use as a secondary effect of an intervention for depression.

 An alternative approach to smoking cessation is through the use of online social networks. Popular components of Web-Assisted Tobacco Interventions (WATIs) are social support networks or discussion boards [24]. The paper by van Mierlo and colleagues [24] provides an analysis of the categories of users who post to discussion boards on WATIs. Similar typographies of users were found from a publicly funded resource (*Smokers Helpline Online*) and from a social enterprise site (*StopSmokingCenter.net*). Of particular interest was the finding that a sub-group (termed “superusers”) who represented less than 1% of registrants accounted for 35-45% of all posts. An earlier review noted the paucity of evidence for the effectiveness of Internet Support Groups [25]. The findings by the van Mierlo team highlight a particular group for specific research attention in the future, given their likely impact on the tone and outcomes of discussion boards.

 Another theme within Internet intervention research is consideration of the target audience; in this special issue, some papers evaluate treatment interventions targeted at symptomatic individuals (see [5, 6, 15]) and others examine the value of public health interventions (see [21, 26]), including in the novel area of online social networks (see [24]). Within Internet-delivered interventions, the major approach remains cognitive behavioral therapy (see [5, 6, 15]), but research is also being conducted with different theoretical approaches, including positive psychology (see [26]). The field is also examining its grounding within various theoretical frameworks, and the paper written by Hilgart and colleagues proposes using a proven methodology (instructional design models) to guide the planning, development, and implementation of Internet interventions [27].

 In conclusion, this theme issue demonstrates the breadth of research being conducted within the *International Society for Research on Internet Interventions*. It shows how rapidly the field is moving forward, pushing beyond initial feasibility and efficacy trials to more nuanced questions, such as the testing of different levels of support, various technologies, potential mechanisms of change, and implementation on a public health scale. The field is well grounded in theoretical models of behavior change, but is calling for more theory-based intervention planning, design, and development. The rich array of research questions and investigations in this special edition shows the field is advancing to not only raising questions for specific populations or specific intervention types, but instead raising research questions for the field as a whole. The papers clearly suggest new lines of research for the future and show the promise of this emerging field.
